# Integrated Assessment of Chemical and Biological Recovery After Diversion and Treatment of Acid Mine Drainage in a Rocky Mountain Stream

**DOI:** 10.1002/etc.5515

**Published:** 2022-12-20

**Authors:** Christopher J. Kotalik, Joseph S. Meyer, Pete Cadmus, James F. Ranville, William H. Clements

**Affiliations:** ^1^ Columbia Environmental Research Laboratory US Geological Survey Columbia Missouri USA; ^2^ Department of Fish, Wildlife and Conservation Biology Colorado State University Fort Collins Colorado USA; ^3^ Department of Chemistry Colorado School of Mines Golden Colorado USA; ^4^ Applied Limnology Professionals Golden Colorado USA; ^5^ Colorado and Wildlife Fort Collins Colorado USA

**Keywords:** Acid mine drainage, benthic ecology, biomonitoring, Superfund site, trace metals

## Abstract

Responses of stream ecosystems to gradual reductions in metal loading following remediation or restoration activities have been well documented in the literature. However, much less is known about how these systems respond to the immediate or more rapid elimination of metal inputs. Construction of a water treatment plant on the North Fork of Clear Creek (NFCC; CO, USA), a US Environmental Protection Agency Superfund site, captured, diverted, and treated the two major point‐source inputs of acid mine drainage (AMD) and provided an opportunity to investigate immediate improvements in water quality. We conducted a 9‐year study that included intensive within‐ and among‐year monitoring of receiving‐stream chemistry and benthic communities before and after construction of the treatment plant. Results showed a 64%–86% decrease in metal concentrations within months at the most contaminated sites. Benthic communities responded with increased abundance and diversity, but downstream stations remained impaired relative to reference conditions, with significantly lower taxonomic richness represented by a few dominant taxa (i.e., *Baetis* sp., *Hydropsyche* sp., *Simulium* sp., Orthocladiinae). Elevated metal concentrations from apparent residual sources, and relatively high conductivity from contributing major ions not removed during the treatment process, are likely limiting downstream recovery. Our study demonstrates that direct AMD treatment can rapidly improve water quality and benefit aquatic life, but effectiveness is limited, in part, to the extent that inputs of metals are captured and treated. Consideration should also be given to the effects of elevated major ion concentrations from the treated effluent not removed during the lime treatment process. Continued chemical and biological monitoring will be needed to quantify the NFCC recovery trajectory and to inform future remediation strategies. *Environ Toxicol Chem* 2023;42:512–524. © 2022 The Authors. *Environmental Toxicology and Chemistry* published by Wiley Periodicals LLC on behalf of SETAC. This article has been contributed to by U.S. Government employees and their work is in the public domain in the USA.

## INTRODUCTION

Remediation and restoration activities conducted in mining‐impacted streams often span several years to decades, leading to gradual recovery after elimination of metals and other stressors (Armitage et al., 2007; Clements et al., 2010; Herbst et al., 2018; Hornberger et al., 2009; Mebane et al., 2015; Runkel et al., 2009; Unruh et al., 2009; Wolff et al., 2019). However, remediation efforts sometimes include rapid elimination of acid mine drainage (AMD) inputs, such as the installation of a water treatment facility or the diversion of mining discharges. With few exceptions (e.g., Leadville Mine Drainage Tunnel on the Arkansas River, CO, USA: Clements et al., 2010; Nelson & Roline, 1996; Junction Creek, Sudbury, ON, Canada: Gunn et al., 2010; and Lake Arnoux, QC, Canada: Mocq et al., 2018), relatively little is known about the recovery of aquatic systems when AMD inputs are rapidly eliminated. More importantly, few of those studies (Clements et al., 2010; Nelson & Roline, 1996) have included extensive, coordinated before–after sampling of chemical and biological conditions at reference and impact sites. Assessing effects of contaminants in systems where water quality improves rapidly allows researchers to better demonstrate causal relationships between stressors and responses. Thus, to better understand the effectiveness of remediation efforts, and to temper expectations for spatial and temporal improvement in receiving waters, more studies are needed of aquatic ecosystems following immediate decreases in metal mining‐related AMD inputs.

Previous long‐term studies of biological recovery in metal mining‐impacted streams have included intensive spatial and temporal sampling. Chadwick et al. (1986) reported gradual increases in abundance and taxonomic richness of benthic macroinvertebrates from 1972 to 1983 in the Silver Bow Creek (MT, USA) after installation of a treatment system to remediate wastewater from a Cu mining district. However, instream concentrations of metals remained elevated after treatment, and high annual variability in macroinvertebrate indices demonstrated the need for multiple years of sampling. Following additional remediation in Silver Bow Creek and the downstream Clark Fork (e.g., removal of tailings), metal concentrations in water and sediments continued decreasing through 2005 (Hornberger et al., 2009). Herbst et al. (2018) also reported gradual increases of abundance and taxonomic richness of benthic macroinvertebrates in streams at the abandoned Leviathan sulfur mine (CA, USA) as a result of a variety of remediation efforts conducted from 1999 through 2009. Similarly, Mebane et al. (2015) reported increases of abundance and taxonomic richness of macroinvertebrates and fishes from 1993 to 2013 in streams at the Co‐ and Cu‐producing Blackbird Mine in east‐central Idaho (USA). High seasonal variability of metal concentrations and relatively high among‐year variability in biological indices during that study demonstrate the need for multiple seasons and multiple years of sampling. In the Upper Arkansas River (CO, USA), remediation efforts resulted in gradual decreases of Cd, Cu, and Zn inputs from a large complex of mines in Leadville that increased abundance and taxonomic richness of benthic macroinvertebrates from 1989 to 2006 (Clements et al., 2010). Similar to results of the studies just described, high annual variability in metal concentrations and biological indices emphasize the need for multiple seasons and multiple years of sampling.

We report the results of a 9‐year AMD‐remediation study that included intensive within‐ and among‐year monitoring of receiving‐stream chemistry and benthic communities before, during, and after construction of a water treatment plant designed to reduce AMD inputs. Unlike most previous studies in which remediation activities occurred over several years, this system is unique because metal loadings were diverted from the receiving stream. As a result, metal concentrations decreased rapidly, providing an opportunity to quantify recovery following the near‐instantaneous elimination of this major stressor. In the present study, we integrate and expand the chemical and biological results previously presented (Cadmus et al., 2016; Kotalik et al., 2021; Lloyd, 2020). A key goal of our study was to compare the rates of chemical and biological recovery in the North Fork of Clear Creek (NFCC; CO, USA) with those of other streams recovering from the long‐term effects of mining impacts where remediation activities occurred over much longer time periods.

## MATERIALS AND METHODS

### Study area

**Figure 1:**
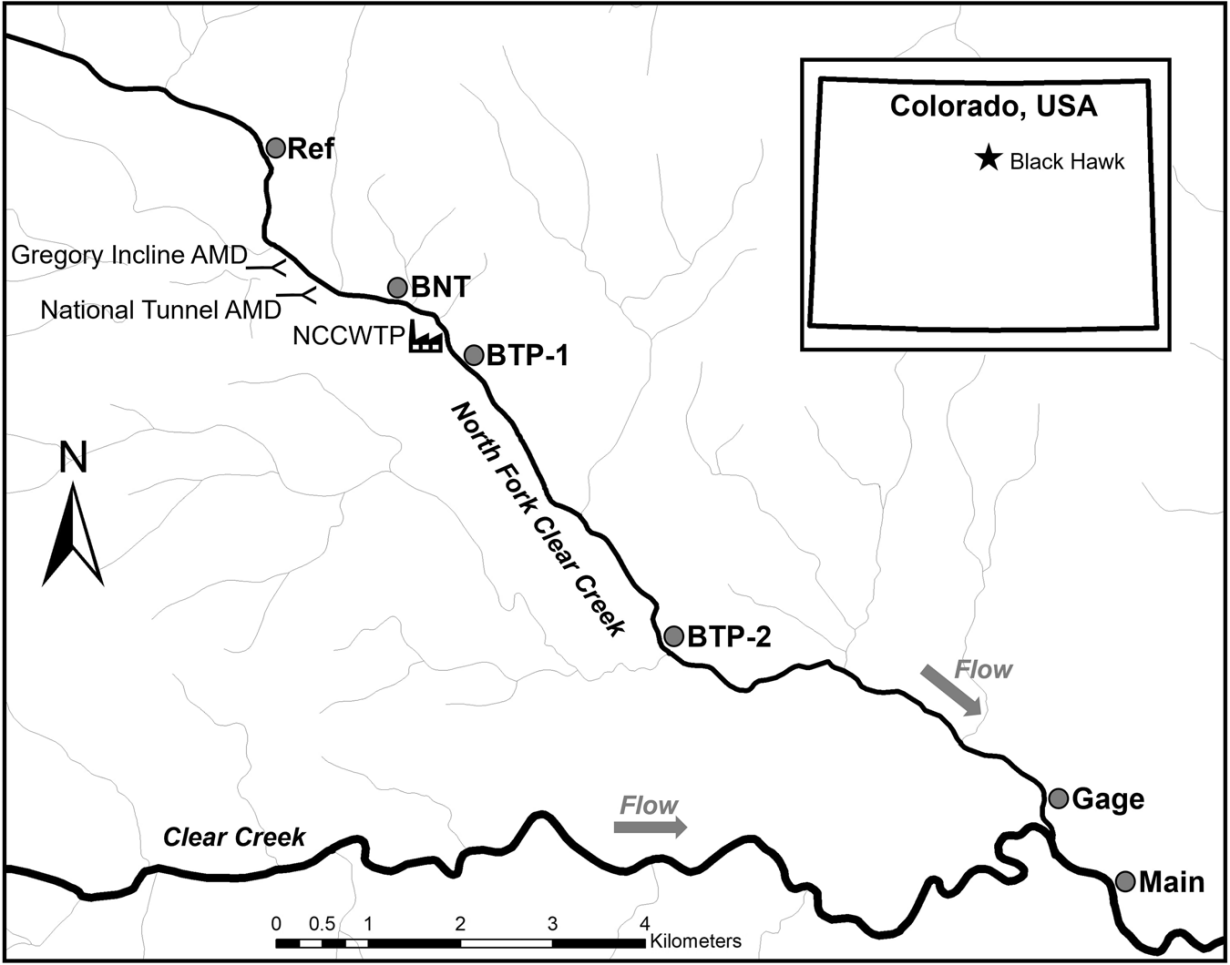
Map of sampling stations located upstream and downstream from the two point‐source inputs of acid mine drainage (AMD) in the North Fork Clear Creek, Colorado, USA. BNT = Below National Tunnel; BTP = Below Treatment Plant; NCCWTP = North Clear Creek Water Teatment Plant.

The NFCC is a third‐order stream located in north‐central Colorado, approximately 50 km west‐northwest of Denver (Figure 1). The stream originates at 3400 m above sea level several km east of the Continental Divide and flows southeast 28 km to its confluence with Clear Creek. The hydrographs of NFCC and Clear Creek are typical of montane streams in the western United States, with low flows during fall and winter and high flows during snowmelt in spring and summer (Supporting Information, Figure S1). Peak discharge (∼2.4 m^3^/s) typically occurs in late May or early June and is approximately 30‐fold higher than the average daily lowest discharge (∼0.08 m^3^/s) in January and February. Historical mining activity resulted in elevated concentrations of several trace elements, significantly affecting aquatic life and potentially threatening human health (Colorado Department of Public Health and Environment [CDPHE], 2017). The two main point‐source inputs of AMD into the NFCC were Gregory Incline and National Tunnel. In 1983, the US Environmental Protection Agency (USEPA) added the Clear Creek–Central City site to the Superfund National Priorities List, with the NFCC from Black Hawk to its confluence with the mainstem of Clear Creek as part of one of the operational units of the Superfund site (CDPHE, 2017). Remediation goals for the NFCC were intended to “support the survival of a reproducing brown trout population in the North Fork of Clear Creek” and “support a viable reproducing brown trout population in the main stem of Clear Creek” (Supporting Information, Table S1; CDPHE & USEPA, 2004).

The USEPA and the CDPHE have undertaken various remedial actions to decrease metal contamination of surface water and associated negative impacts to aquatic species, including removal of waste‐rock piles and construction of a water treatment plant (CDPHE, 2017). The North Clear Creek Water Treatment Plant (NCCWTP) began operating in spring 2017 and was designed to treat AMD entering the NFCC from Gregory Gulch, Gregory Incline, and National Tunnel. The Gregory Incline and National Tunnel waters are diverted around a 1.7‐km stream segment and piped directly to the NCCWTP, where the treated water is discharged (4.3–10.3 L/s) into the NFCC. Thus, diversion and water treatment have altered the water chemistry in the NFCC upstream of the NCCWTP to Gregory Incline (lower metal concentrations, water hardness, sulfate, and conductivity) and downstream of the NCCWTP to its confluence with Clear Creek (lower metal concentrations, but higher water hardness and conductivity).

#### Sampling stations

Water quality and benthic communities were measured at six sampling locations on the NFCC and Clear Creek (Figure 1 and Supporting Information, Table S2). The reference station was upstream of the two point‐source AMD inputs that discharged directly into the NFCC before the water treatment plant became operational in summer 2017. Immediately upstream from the NCCWTP, station Below National Tunnel (BNT) was exposed to both AMD sources before remediation; however, after remediation these AMD sources were diverted away from BNT directly to the treatment facility. The stations BTP‐1 and BTP‐2 (Below Treatment Plant) were located downstream of the treatment plant and received treated AMD effluent. Farther downstream, station Gage was located at a US Geological Survey gaging station (USGS 06718550) and received treated AMD effluent as well as discharge from the Black Hawk–Central City Sanitation District Wastewater Treatment Facility. Finally, station Main was located on the mainstem of Clear Creek downstream of the confluence with the NFCC.

During base flow, stream reaches among all sampling stations on the NFCC were less than 0.5‐m water depth, with approximately 2‐ to 4‐m wetted channel stream width. The substrate consisted primarily of sand, pebble, and cobble in typical riffle–pool–run sequences. Streamside riparian vegetation was dominated by willow (*Salix* spp.) and narrowleaf cottonwood (*Populus angustifolia*), with varying amounts of riparian shading. Water depth at station Main was less than 1 m, with approximately 10‐ to 15‐m wetted channel stream width. The substrate consisted primarily of pebble, cobble, and boulder substrate, and streamside vegetation was dominated by the same species as the NFCC sites, with relatively little riparian shading.

#### Water sampling and chemical analyses

Water samples were collected for chemical analyses at the stations on the NFCC and Clear Creek from November 2011 to October 2019. Samples were generally collected in late spring and fall; however, to better characterize changes in water chemistry after the treatment facility became operational in 2017, we collected samples for chemical analyses approximately every 4 weeks during low flow (August–April) and approximately every 8 weeks during high flow (May–July). In the field, we measured temperature, pH, conductivity, alkalinity, and ferrous iron (Fe^2+^). Nonfiltered and filtered water samples were collected for determination of total‐ and dissolved‐metal concentrations by inductively coupled plasma–atomic emission spectroscopy. Water samples for analysis of total and dissolved organic carbon (DOC) were collected in amber bottles and analyzed by combustion (Shimadzu model TOC‐L). Filtered water samples were also analyzed for major inorganic ions by ion chromatography. Deionized water was identically processed to provide method blanks. Details of the collection, preservation, storage, and analytical procedures are presented in Meyer et al. (2022). In addition, we obtained long‐term water quality data from the Department of Public Works and Utilities in the City of Westminster, Colorado (TechLaw 2013). Those data included concentrations of total and dissolved metals at the Gage sampling site from October 1994 to February 2017. Details of the collection, preservation, storage, and analytical procedures for these samples are presented in TechLaw (2013).

#### Biological sampling

Benthic macroinvertebrate samples were collected seasonally (generally early May and mid‐October) from 2011 through 2019 at all stations except for BTP‐1, which was added in 2017 to better characterize postremediation responses to changes in water quality. In addition, we collected samples monthly during the summers (July–September) of 2017 and 2018 at all stations except Main. Details of benthic sampling and preservation procedures are presented in Kotalik et al. (2021). In the laboratory, invertebrates were identified to the lowest taxonomic resolution possible, typically to genus (except for chironomids, which were identified to subfamily).

#### Data analyses

Because we measured water quality with much greater frequency than benthic macroinvertebrates, we composited water quality samples to provide a more robust integration of physicochemical and biological data. The structure of benthic communities on any given date is likely influenced by water quality characteristics that occur prior to that sampling occasion. Although it is difficult to know exactly the appropriate time window and the relative importance of exposures on any given date, we calculated composite water chemistry for each biological‐sampling date using a single set of rules (see the Supporting Information). Briefly, water samples were classified as Spring (January–May), Summer (July–September), or Fall (October–November). Composite water chemistry was calculated from all eligible water chemistry data for a benthic‐sampling event up to 3 months before (depending on season), and up to 1 week after.

To develop an index of the potential risk to aquatic communities from exposure to the mixture of metals, we calculated chronic criteria units (CUs) for each of the seven metals included in the current analysis (Al, Cd, Cu, Fe, Mn, Ni, and Zn). We then summed the CUs of the metals to produce chronic cumulative criteria units (CCUs). For these calculations we used a modification of the calculation procedure in Clements et al. (2021). Details of the algorithm used to estimate individual CUs are in the Supporting Information.

All parametric statistical analyses were conducted using SAS Ver 9.3 (SAS Institute). Effects of station, season (spring, summer, fall), and remediation (before vs. after) on benthic macroinvertebrate communities were analyzed using three‐way analysis of variance (ANOVA; using PROC general linear model [GLM; SAS Institute]). Before statistical analyses, benthic and water chemistry data were averaged for BTP‐1 and BTP‐2 as a combined BTP station because pretreatment data were not available for BTP‐1 and because these two stations have very similar water chemistry characteristics (Meyer et al., 2022). To generate BTP station averages, benthic abundances among taxa were averaged for every paired BTP‐1 and BTP‐2 sampling date, and the composite water chemistry values assigned to BTP‐1 and BTP‐2 for each respective benthic sampling date were averaged. To quantify the effectiveness of remediation, we tested for a significant interaction between station and remediation treatment. We were particularly interested in determining whether differences between the reference and downstream stations were greater before remediation than after, providing support for our hypothesis that AMD treatment improved water quality and macroinvertebrate communities. Abundance and water chemistry data were log‐transformed (ln + 1) to satisfy assumptions of parametric statistics. If the overall model was significant, we used least square means to test for significant differences among individual stations, among seasons, and between remediation treatments.

To identify the major physicochemical variables that were responsible for the observed differences among stations, treatments, and seasons, we used a GLM procedure (PROC GLMSELECT) to estimate the influence of water quality on three community metrics: number of taxa, total abundance, and abundance of Heptageniidae. Abundance of heptageniid mayflies was included because these organisms are especially sensitive to metals (Clements et al., 2010; Mebane et al., 2015). Models were developed using a stepwise forward‐selection procedure, and the best models were identified based on Akaike's information criterion (AIC) values. One important goal of our analysis was to determine the amount of variation that could be explained based only on physicochemical variables and to compare these results with the categorical analyses just described.

Multivariate analysis of benthic communities was conducted using PRIMER‐e Ver 7 + permutational multivariate ANOVA (Quest Research; Anderson et al., 2008). Replicate macroinvertebrate samples (*n* = 5) were averaged for each site‐date observation and square‐root transformed, and then Bray–Curtis distance matrices were calculated. Community composition was based on the abundance of the 47 taxa that were present in 5% or more of all benthic samples from 2011 to 2019. We used nonmetric multidimensional scaling (NMDS) ordination to visualize differences in benthic community composition among stations before and after AMD remediation. To determine the influence of water chemistry on differences in benthic community composition in ordination space, superimposed vectors of physiochemical variables (based on Pearson linear correlations) were overlaid on the NMDS plot. Before vectorization, we log‐transformed and normalized the water chemistry data as (*x*_i_ – µ)/σ, where *x*_i_ is the observed value, μ is the mean value, and σ is the standard deviation. The direction of these vectors indicates the direction a variable increased, and the length of these vectors indicates the strength of their correlation with the NMDS axes.

## RESULTS

### Water chemistry

**Figure 2:**
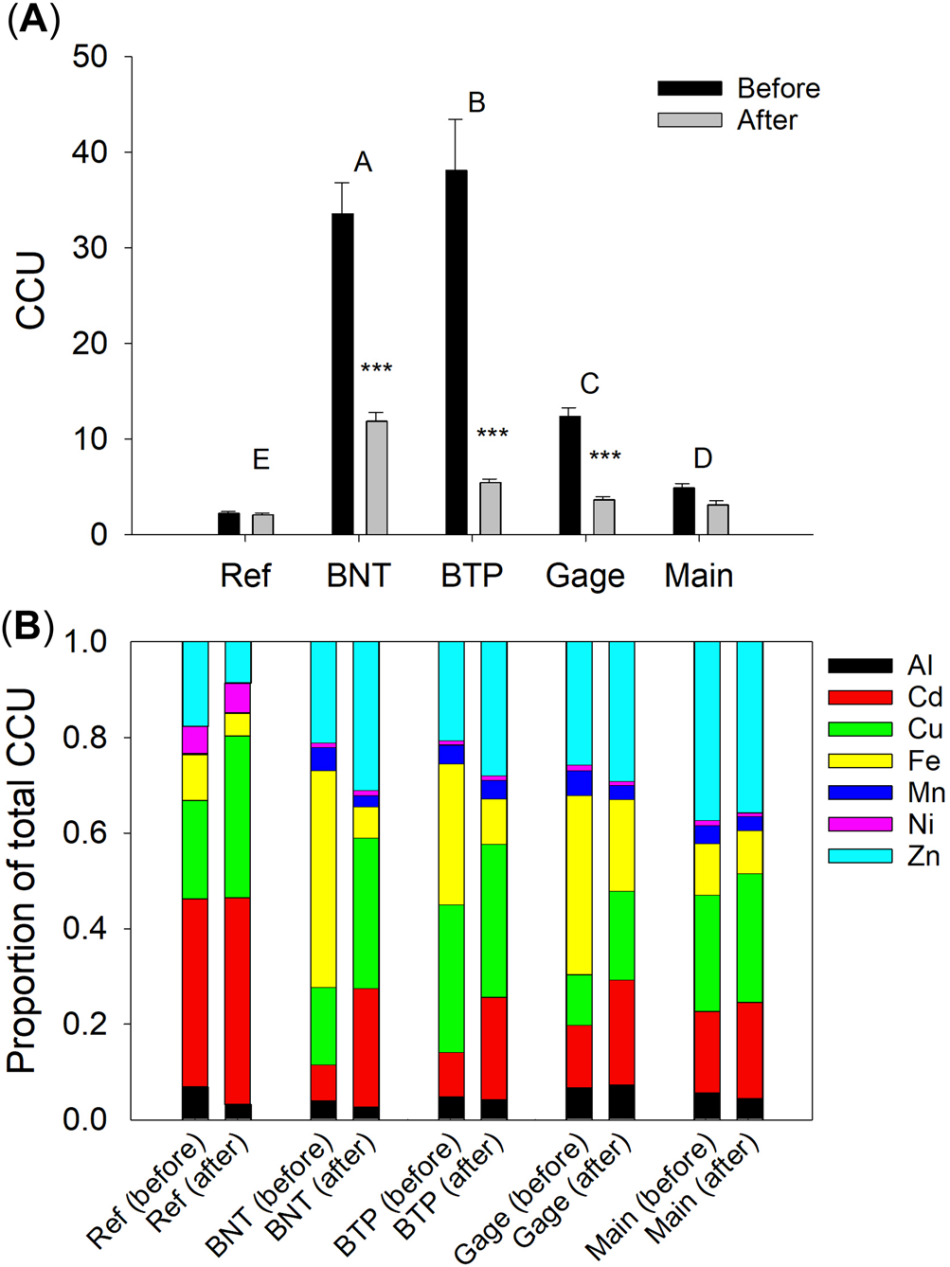
(**A**) Spatiotemporal variation in mean (±SE) metal concentrations (as chronic cumulative criteria units [CCUs]) at North Fork Clear Creek (NFCC) showing differences among stations and between treatments (before vs. after remediation). Stations with the same letter are not significantly different based on least square means tests. Asterisks indicate significant effects of remediation at each station (***p < 0.001). (**B**) Relative contribution of metals to the CCU at NFCC stations before and after remediation. BNT = Below National Tunnel; BTP = Below Treatment Plant.

Metal concentrations (expressed as CCUs) were significantly elevated at all stations downstream from the AMD inputs to the NFCC (Figure 2 and Supporting Information, Tables S3 and S4). Before remediation, CCUs at stations immediately downstream from the AMD inputs (BNT and BTP) were 15‐ to 17‐fold greater than at the reference station. Metal concentrations decreased downstream, but remained significantly elevated even at the farthest downstream station (Main). The CCUs at the most contaminated stations (BNT, BTP, and Gage) were significantly lower after remediation, decreasing by 64%–86%. Spatial variation in metal concentrations and the responses to remediation were generally consistent among metals (Supporting Information, Figure S2). Concentrations of all of the metals we measured decreased 1) as downstream distance from the historical AMD inputs increased, and 2) after remediation.

The relative contributions of individual metals to the CCUs varied spatially and based on remediation treatment (Figure 2). Before remediation, Cu, Fe, and Zn contributed most to the total CCUs at the downstream sites. The relative contribution of Fe to the CCUs was much lower after remediation, especially at BNT and BTP.

**Figure 3:**
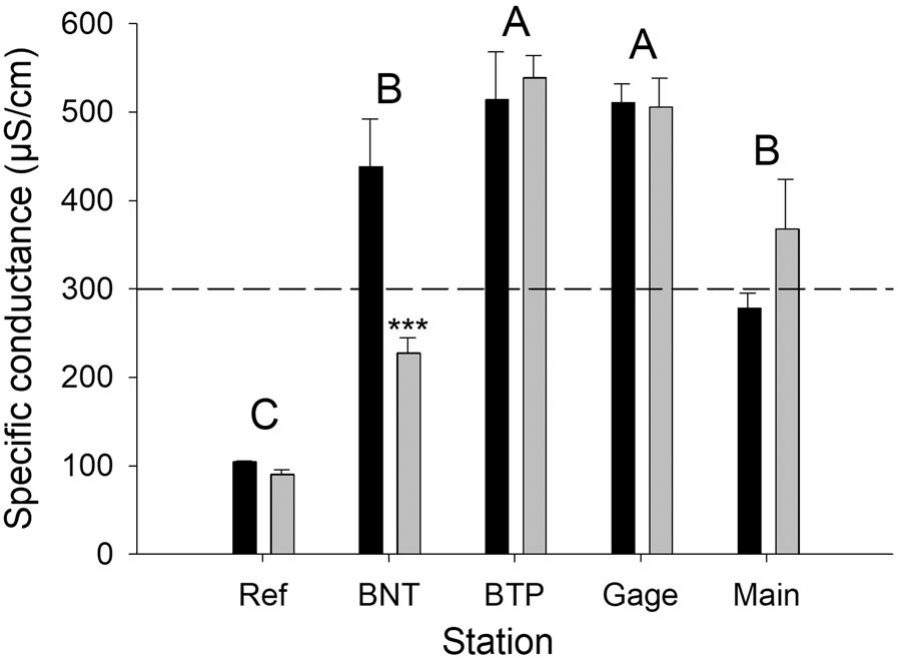
Spatiotemporal variation in mean (±SE) specific conductance (µS/cm) at North Fork Clear Creek showing differences among stations and between treatments before (black bars) versus after (gray bars) remediation. Stations with the same letter are not significantly different based on least square means tests. The dashed line indicates the 300‐µS/cm benchmark established by the US Environmental Protection Agency (Comier et al. 2013) for headwater streams. Asterisks indicate significant effects of remediation at each station (***p < 0.001). BNT = Below National Tunnel; BTP = Below Treatment Plant.

In addition to metal concentrations, several other physicochemical characteristics that we measured (e.g., pH, water hardness, alkalinity, specific conductance, major cations and anions) varied among stations and were affected by remediation (Supporting Information, Figure S3, Table S5). In particular, specific conductance (Figure 3 and Supporting Information, Table S5) was significantly elevated at stations located downstream of the NCCWTP (BTP, Gage, Main) and was above the 300‐µS/cm benchmark established by the USEPA (Cormier et al. 2013) for headwater streams. Specific conductance was significantly decreased at station BNT after remediation, but it remained elevated at all stations downstream of the water treatment facility.

#### Spatiotemporal variation in benthic communities

**Figure 4:**
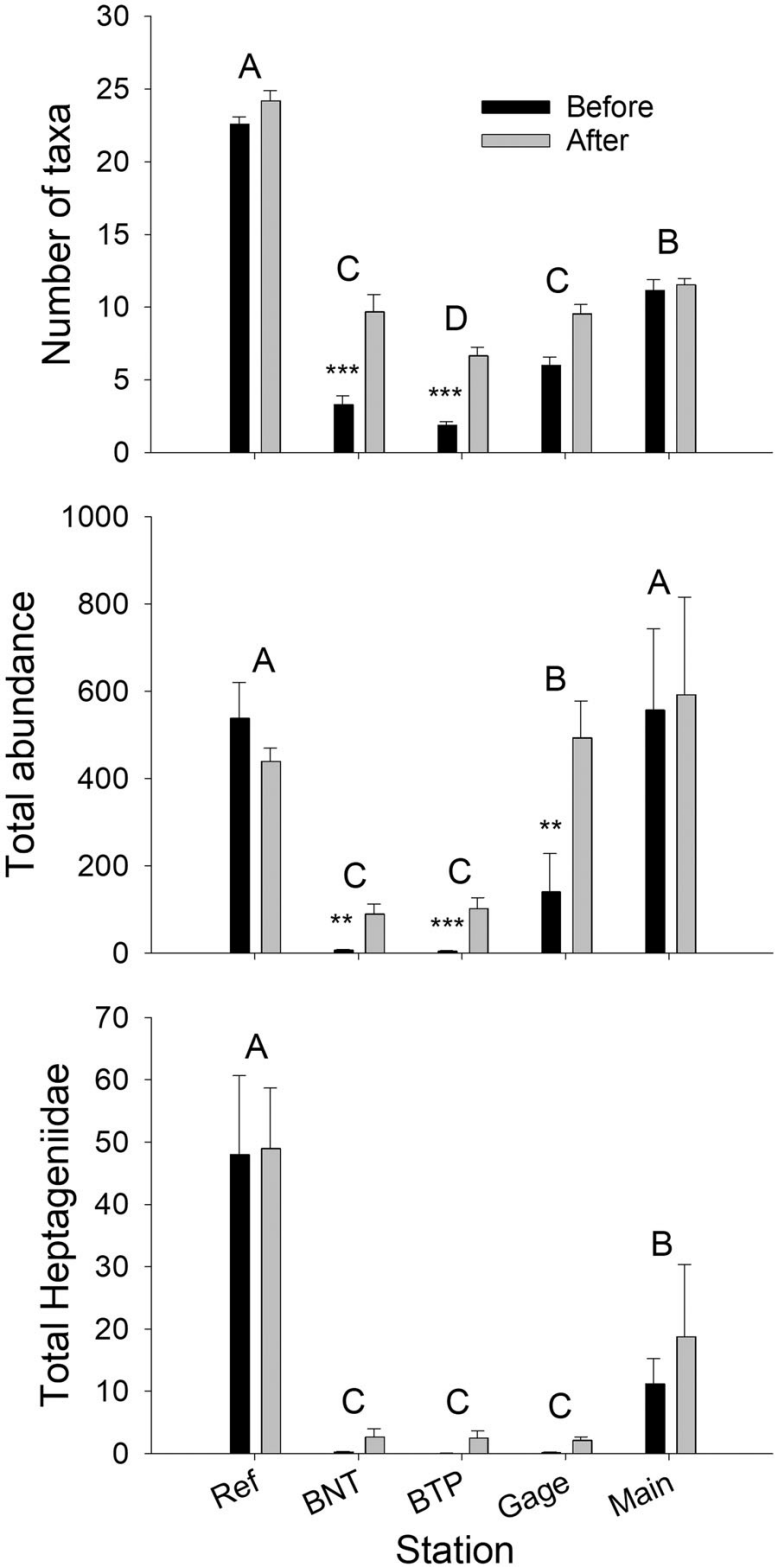
Spatiotemporal variation in mean (±SE) benthic community metrics at North Fork Clear Creek showing differences among stations and between treatments (before vs. after remediation). Stations with the same letter are not significantly different based on least square means tests. Asterisks indicate significant effects of remediation at each station (**p < 0.01; ***p < 0.001). BNT = Below National Tunnel; BTP = Below Treatment Plant.

Macroinvertebrate communities varied significantly among stations, seasons, and restoration treatments (before vs. after remediation) in the NFCC (Figure 4 and Table 1). The variables station, season, remediation treatment, and the station × remediation treatment interaction term explained 76%–90% of the total variation in the three community metrics that we examined. Each of these metrics was significantly reduced downstream from the source of metals, and each metric progressively increased as distance downstream increased (Supporting Information, Tables S3 and S4); however, the degree of recovery and the location where recovery occurred varied among metrics. Although the mean number of taxa significantly increased downstream, it remained approximately 50% lower at the farthest downstream station (Main) compared with the reference station. Total macroinvertebrate abundance was the most improved community metric, especially after remediation, and was not significantly different from reference station values at the farthest downstream station. In contrast, abundance of metal‐sensitive heptageniid mayflies showed little change until the most downstream station, where it still remained approximately 70% below the reference abundance. Finally, the significant variation in total macroinvertebrate abundance among seasons resulted from lower abundance in spring compared with summer and fall (Supporting Information, Table S4).

**Table 1:** Results of three‐way analysis of variance testing for the effects of station, season, and remediation treatment (before vs. after), and the station × treatment interaction term for macroinvertebrate community metrics, metal concentrations (as chronic cumulative criterion units [CCUs]), and specific conductance measured in the North Fork of Clear Creek, CO

	Overall model			Source of variation		
Variable	*F*‐value	*p*‐value	*R* ^2^		*F*‐value	*p*‐value
Number of taxa	89.79	0.0001	0.90	Station	232	0.0001
				Season	1.1	0.3459
				Remediation	27.8	0.0001
				Station × Remediation	4.1	0.0038
Total abundance	33.59	0.0001	0.77	Station	69.4	0.0001
				Season	5.4	0.0061
				Remediation	26.4	0.0001
				Station × Remediation	7.6	0.0001
Total Heptageniidae	31.09	0.0001	0.76	Station	82.1	0.0001
				Season	1.9	0.1566
				Remediation	5.9	0.0167
				Station × Remediation	0.5	0.7191
Metal concentration	102.51	0.0001	0.91	Station	192.8	0.0001
(CCU)				Season	2.4	0.0980
				Remediation	177.5	0.0001
				Station × Remediation	30.3	0.0001
Specific conductance	79.69	0.0001	0.89	Station	195	0.0001
				Season	13.8	0.0001
				Remediation	0.1	0.7345
				Station × Remediation	8.5	0.0001

The table shows overall model results, *R* ^2^ values, and significance of the individual factors in each model.

Across all stations, each of the community metrics increased after remediation, as indicated by the significant remediation treatment effects in these ANOVA models (Table 1). The station × remediation treatment interaction terms were also highly significant for total abundance and number of taxa, indicating that increases after remediation were greater at downstream stations. These findings provide support for our hypothesis that improvements in abundance and number of taxa were a direct result of remediation. In contrast, the interaction between station and treatment was not significant for abundance of Heptageniidae, likely because of relatively high variability of this metric and because increases in abundance after remediation were relatively modest.

**Figure 5:**
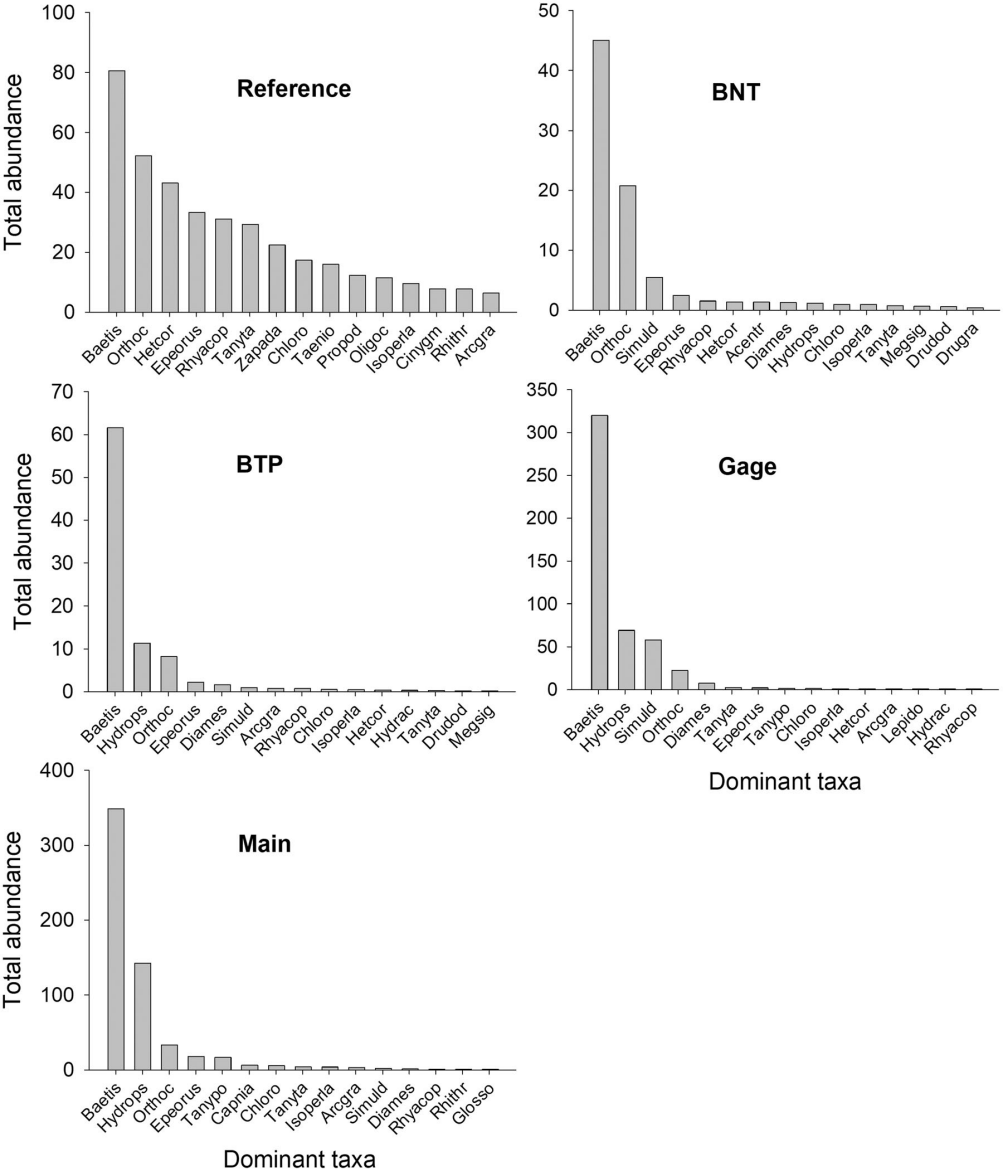
Ranked abundance of the 15 dominant taxa measured at each of the five North Fork Clear Creek sampling stations after remediation treatments. See the Supporting Information, Table S6, for full taxonomic descriptions associated with abbreviations. BNT = Below National Tunnel; BTP = Below Treatment Plant.

Although macroinvertebrate community metrics improved after remediation, the ranked abundance of dominant taxa remained very different between reference and downstream stations (Figure 5). In contrast to the reference station, abundance distributions at all downstream stations were highly skewed and dominated by one or two taxa, which together accounted for 74%–83% of the entire community. Baetid mayflies were the dominant organisms at all stations, but their relative contributions to total abundance was much greater at downstream stations.

The GLMs developed using physicochemical predictors explained approximately the same amount of variation as those based on the categorical ANOVA analyses just described (Table 2). The best models for the benthic community metrics consistently included metal concentration (expressed as CCUs of a three‐metal mixture of Cd, Cu, and Zn) and sulfate (SO_4_) as the most important predictors. Although DOC concentration, pH, and alkalinity were included in some models, their relative importance varied among metrics. Based on the “rule of thumb” for interpreting AIC values among competing models (Burnham & Anderson, 2004), there was strong support for models that included metals and SO_4_ as predictors of total macroinvertebrate abundance and abundance of heptageniids, but generally less support for models that also included pH and DOC. These results indicate that variation in benthic communities among stations, seasons, and remediation treatments was determined primarily by concentrations of metals and SO_4_.

**Table 2:** Results of general linear model selection for macroinvertebrate community responses to metals in the North Fork of Clear Creek, CO

	Model selection results						Analysis of variance		
Community metric	Step	Parameter	AIC	Parameter estimate	*t*‐value	*p*‐value	*F*‐value	*p*‐value	*R* ^2^
Number of taxa	0	Intercept	612.1	54.1	9.83	0.0001	248.4	<0.0001	0.90
	1	SO_4_	452.1	−4.22	−17.72	0.0001			
	2	CCU	370.9	−4.66	−11.06	0.0001			
	3	DOC	353.7	−1.55	−4.08	0.0001			
	4	pH	346.9	−2.04	−2.97	0.0037			
Total abundance	0	Intercept	285	8.07	16.28	0.0001	84.5	<0.0001	0.74
	1	CCU	163.1	−1.18	−8.19	0.0001			
	2	Alkalinity	151.5	0.06	6.26	0.0001			
	3	SO_4_	139.6	−0.44	−4.32	0.0001			
	4	DOC	127.9	−0.61	−3.72	0.0003			
Total Heptageniidae	0	Intercept	223.6	11.34	6.41	0.0001	79.1	<0.0001	0.73
	1	SO_4_	116.7	−0.76	−9.89	0.0001			
	2	CCU	81.6	−0.97	−7.12	0.0001			
	3	pH	73	−0.66	−2.98	0.0036			
	4	DOC	72.4	−0.2	−1.59	0.1144			

The table shows the overall analysis of variance results for the best models, *R* ^2^ values, environmental variables included in these models, parameter estimates, and their statistical significance. Variables were selected based on AIC values whereby the best models are defined as those with the lowest AIC values.

AIC = Akaike's information criterion; CCU = chronic cumulative criteria unit; DOC = dissolved organic carbon.

**Figure 6:**
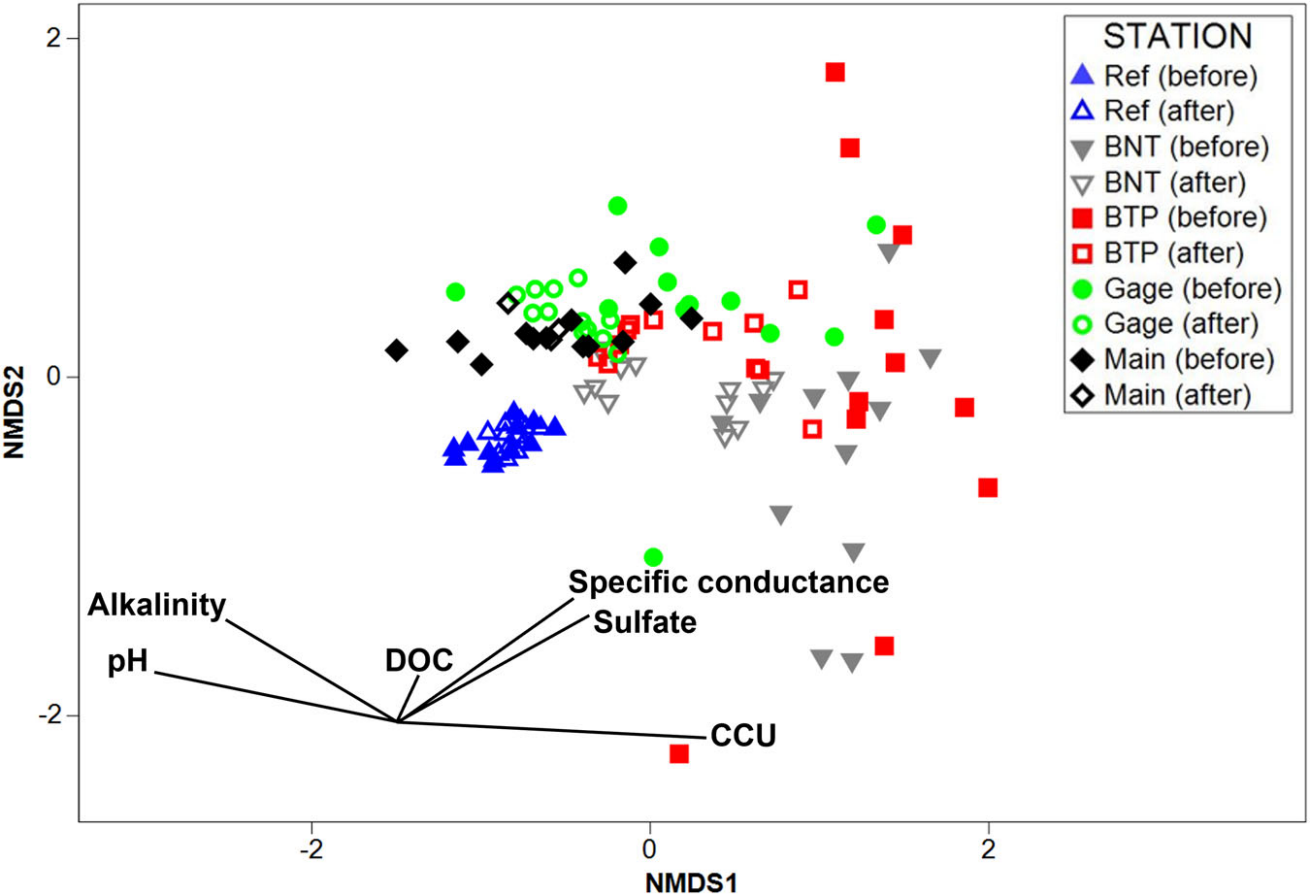
Results of the nonmetric multidimensional scaling (NMDS) ordination showing separation in benthic community composition among stations in North Fork Clear Creek before and after remediation. The figure also shows the physicochemical variables linearly correlated with the 2‐day ordination plot. The direction of a vector indicates the direction that a variable increases, and the length indicates the strength of the variable's correlation with the NMDS axis. DOC = dissolved organic carbon; CCU = chronic cumulative criteria units. BNT = Below National Tunnel; BTP = Below Treatment Plant.

In the NMDS analysis, the reference station was distinctly separated from all other stations before and after remediation (Figure 6). The overall tight clustering of the reference ordinations demonstrates compositional stability at this station. Before remediation, stations BNT, BTP, and Gage were the most dissimilar compared with reference, and benthic community composition among these stations was highly variable. After treatment, these stations tended to ordinate closer to reference and were more compositionally consistent. This suggests that the benthic composition of these downstream stations became more similar to that observed at the upstream reference station. The overlaid vectors alkalinity, pH, and CCU were highly correlated with the NMDS1 axis, whereas DOC was weakly correlated with the NMDS2 axis; specific conductance and SO_4_ were moderately correlated with both NMDS axes. The CCU variable was inversely correlated with alkalinity and pH on the NMDS1 axis. The ordinations of downstream stations shifted along the NMDS1 axis after treatment because of changes in water chemistry. In other words, as CCU decreased and pH and alkalinity increased, the downstream benthic communities became more similar to the reference.

## DISCUSSION

### Improvements in water quality after remediation

Remediation of two major AMD inputs into the NFCC significantly decreased metal concentrations at all downstream stations within the first year after the water treatment plant began removing metals. The greatest improvements were at the two most heavily contaminated stations (BNT and BTP); however, the CCUs also decreased considerably at Gage and in the mainstem of Clear Creek after remediation. Because the effluents from Gregory Incline and National Tunnel, the two major sources of metals to the NFCC, were diverted around station BNT, we expected the greatest and most rapid improvements in water quality at this station. Although mean CCUs at BNT decreased by approximately 65% after diversion of these effluents (mean postremediation CCUs = 11.9), they were greater than at other downstream stations and remained elevated above expected safe concentrations. Thus, it is likely that other sources of metals contributed to the residual elevated concentrations at BNT (Meyer et al., 2022).

Previous studies have used CCUs to quantify potential effects of metal mixtures on benthic communities in the field (Clements et al., 2000; Herbst et al., 2018; Mebane et al., 2015) and in stream mesocosm experiments (Clements et al., 2019; Mebane et al., 2020). Important limitations of this approach include 1) the assumption that metal effects do not interact synergistically or antagonistically (Meyer et al., 2015), and 2) the effects of the same CCU value might differ depending on the metals in the mixture and their relative proportions. Synergistic and antagonistic interactions among metals have been measured in laboratory toxicity tests (see Meyer et al., 2015). However, because of logistical challenges, verifying the additive toxicity of individual CUs on benthic communities is more difficult. In some mesocosm experiments, the assumption of equivalent toxicity of individual CUs has been supported (Mebane et al., 2020); but in other experiments, CUs including Cu or Fe, either alone or in combination with other metals, were more toxic to aquatic insects than those including Zn alone (Clements et al., 2019). Differences in the relative toxicity of individual metals (when expressed as CUs) to the toxicity of the summed CCUs could result from several factors, including potential metal interactions, inaccurate criteria values, or failure to account for physical effects of metals such as Fe (Cadmus et al., 2016; Linton et al., 2007).

In addition to the significant spatial variation in concentrations of metals in the NFCC, their relative contributions to the metal mixture at the various sites changed considerably after water treatment (Figure 2B). However, despite changes in the relative contributions of individual metals to the total CCUs, postremediation variation among the downstream stations was minimal. Thus, differences in the relative contributions of metals to the CCUs probably did not affect downstream responses of benthic communities.

As an example of changes in the composition of the metal mixtures, the percentage contributions of Fe to the total CCUs greatly decreased after remediation, whereas the contributions of Cd and Cu increased (Figure 2B). The inverse relationship between Cu and Fe is toxicologically important, because 1) mesocosm experiments have shown that Cu and Fe are responsible for much of the toxicity to benthic organisms relative to their aquatic life criterion values (Clements et al., 2019), and 2) their concentrations in surface waters are regulated in the United States and most other jurisdictions around the world. In the NFCC, the percentage of Cu that is dissolved is strongly and inversely correlated with the particulate Fe concentration (Meyer et al., 2022). This relationship in part explains why the concentration of dissolved Cu at Gage decreased by only 20% after treatment whereas the total Cu concentration decreased by 67% (Meyer et al., 2022). Therefore, although moderate concentrations (>250 mg/L) of particulate Fe can impair benthic communities by deposition of Fe oxyhydroxide precipitates (Cadmus et al., 2016), particulate Fe can also sorb some trace metals and thereby decrease their dissolved concentration.

### Spatial and temporal changes in benthic community composition

We compared two different statistical approaches to quantify the effects of station, season, restoration treatment, and physicochemical variables on benthic community metrics. Analysis of variance is a typical approach used in before–after–control–impact (BACI) study designs whereby the significance of an interaction term is used to test the hypothesis that differences between control and impacted sites were the direct result of an impact, usually a planned or unplanned perturbation (Chevalier et al., 2019; Stewart‐Oaten et al., 1986). This approach provides a much stronger causal link between stressors and responses than the more typical before versus after or control versus impact study designs. In the NFCC we used a BACI design to determine whether differences between upstream reference and downstream metal‐impacted sites were a result of reduced metal concentrations. Because benthic communities at the reference site did not change after remediation, the increases in abundance and number of taxa at the downstream sites can be directly linked to improvements in water quality. In contrast, the lack of a significant interaction term for heptageniid mayflies indicated relatively little response of these organisms to decreased metal concentrations.

Our second statistical analysis used a model‐selection approach to identify the major physicochemical variables responsible for changes in macroinvertebrate metrics. The amount of variation explained by these models (73%–90%) was similar to that based on the categorical BACI analyses. Spatial and temporal variation in each benthic metric could be explained by a relatively simple model that included only four variables. Model results for these three community metrics were consistent with results of the NMDS analyses that identified CCU, SO_4_, and conductivity as the most important predictors of benthic community composition. These results demonstrate that the differences in macroinvertebrate communities among stations, seasons, and restoration treatments were strongly influenced by water chemistry.

Although the composition of benthic communities at reference and downstream sites was quite different after remediation, the mayfly genus *Baetis* was the dominant taxon at all sites. Previous studies have classified baetids as relatively tolerant to metals and other anthropogenic stressors based on their presence in disturbed streams (Cain et al., 2004; Clements et al., 2000; Iwasaki et al., 2012; Maret et al., 2003). However, stream mesocosm experiments conducted with metals or major ions have demonstrated that these organisms are actually quite sensitive to these stressors (Cadmus et al., 2016; Cadmus et al., 2019; Clements & Kotalik, 2016). Our leading hypothesis for the inconsistency between the field and mesocosm results is the rapid colonization propensity of baetids, either by downstream drift or by adults ovipositing eggs upstream. Baetids are often the dominant organisms in drift samples (Mackay, 1992; Rader, 1997), and despite their sensitivity to dissolved metals, are often among the first groups to recover after remediation (Clements et al., 2021; Mebane et al., 2015). We speculate that the extremely high density of baetids at the Gage site, which was 4‐ to 6‐fold greater than at any other site on the NFCC (including the reference site), was likely a result of its close proximity to the mainstem of Clear Creek. The 0.4‐km upstream flight of adult baetids, as well as upstream colonization by swimming larval baetids from Clear Creek to Gage, likely contributed most or all of the recolonization at this station. Further upstream, reduced baetid survival due to relatively higher metal concentrations, and fewer numbers of adults sourced from Clear Creek, may account for the much lower densities of baetids at BTP compared with Gage.

In contrast to baetids, hydropsychid caddisflies are highly tolerant of metals and often abundant in metal‐contaminated streams (Cain et al., 2004; Clements et al., 2000). In the NFCC, hydropsychids were the second most abundant taxon at the two farthest downstream sites (BTP and Gage). Because hydropsychids occurred at very low densities at the upstream reference site and have relatively low drift propensity (Rader, 1997), it is unlikely that recolonization of these organisms occurred by downstream drift. Caddisflies metamorphose to winged adults to reproduce, and they have a propensity to fly upstream (Winterbourn & Crowe, 2001). The presence of hydropsychids at these sites was likely due to upstream flight from Clear Creek and subsequent oviposition by adults. These results demonstrate the importance of considering life‐history characteristics and patterns of dispersal and recolonization of dominant taxa when evaluating or predicting the recovery of disturbed streams. Although recovery of benthic macroinvertebrates after a disturbance is often attributed to drift from upstream reference communities (Williams & Hynes, 1976), our results suggest that upstream movement of winged adults is also an important driver for recolonization for some taxa.

### Limited improvements in benthic communities after remediation

In previous long‐term studies of mining‐impacted streams, benthic communities gradually improved after remediation of metal contamination (Clements et al., 2010; Herbst et al., 2018; Mebane et al., 2015). In the NFCC, total macroinvertebrate abundance, number of taxa, and abundance of metal‐sensitive heptageniid mayflies significantly increased after remediation, but these improvements were relatively modest and varied among stations and metrics. Despite improvements in water quality at downstream stations, all of the metrics remained significantly lower for most downstream sites compared with the upstream reference station. In addition, ranked abundance distributions of dominant taxa at all downstream stations differed considerably from the reference site. Although total macroinvertebrate abundance at the two farthest downstream stations was similar to abundance at the reference site after remediation, these downstream stations were dominated by only one or two taxa. The limited improvement in benthic communities was especially notable at BNT and BTP, the most heavily impacted stations. The modest recovery of NFCC 2 years after remediation was unexpected given that metal concentrations rapidly decreased soon after the NCCWTP became operational (Meyer et al., 2022). This result was particularly surprising at BNT because of its proximity to the upstream reference station and because discharges from the two major AMD sources (Gregory Incline and National Tunnel) were completely diverted around this station. We have several hypotheses to explain these results. It is possible that only 2 years after remediation is an insufficient amount of time for recovery of benthic communities in the NFCC. However, recent data collected from BNT‐1 and BNT‐2 showed no evidence of further recovery. Mean macroinvertebrate abundance at this station collected over four sampling occasions in 2020 and 2021 was 30.0 (±8.3) and 15.2 (±3.1), respectively (C. Kotalik, unpublished data). Clements et al. (2021) reported that mean recovery times across four western streams was approximately 10 years after remediation was initiated. However, it is important to note that significant improvement in benthic communities was observed in these systems immediately after remediation began. Also, unlike the NFCC, remediation in those systems generally resulted in more gradual decreases in metal concentrations because metal discharges were not diverted around the impacted stations. When recovery observed at the NFCC is compared with the East Fork of the Arkansas River, a similar size stream in which metal concentrations also immediately decreased after remediation, macroinvertebrate abundance increased approximately five‐fold within 6 months (Clements et al., 2010). Although 2 years is likely an insufficient length of time for the NFCC to fully recover, we expected much greater improvement at BNT given that it was only 3.5 km downstream of an upstream source of recolonization, and the two AMD effluents were diverted around it.

Recovery of stream ecosystems after either an episodic or chronic disturbance is determined by biotic and abiotic factors that operate across landscape scales (Clements et al., 2021; Lepori et al., 2005; Niemi et al., 1990). Factors such as the frequency and severity of the perturbation, hydrologic variability, lack of an upstream source of recolonization, the establishment of novel communities, and the presence of residual stressors (e.g., degraded habitat) can delay recovery of lotic ecosystems. In the NFCC, benthic communities were severely degraded by metals and poor benthic habitat (e.g., high embeddedness, low interstitial space) before remediation. For example, total macroinvertebrate abundance at stations BNT and BTP was approximately 100‐fold lower than at the reference site. However, studies conducted in other mining‐impacted streams with similar preremediation metal concentrations and equally impoverished benthic communities recovered rapidly after metal concentrations decreased (Herbst et al., 2018; Mebane et al., 2015). Therefore, we do not believe that failure of this system to recover was a result of severity of the metal contamination before remediation; instead, other residual stressors, such as degraded habitat, may be contributing to the lack of recovery.

Hydrologic variability can also influence the recovery of streams from disturbance. Hydrologic conditions in the NFCC during our study were highly variable, with summer convection storms capable of increasing stream discharge by 10‐ to 50‐fold over several days (Supporting Information, Figure S1). Although it is possible that high discharge might have slowed recovery in the NFCC by disturbing recently recolonized substrate, theoretical and empirical research suggests that hydrologic variability could have the opposite effect. Clements et al. (2021) speculated that hydrologic variability selected for taxa capable of rapid recolonization and thus might increase the rate of recovery. In stream mesocosm experiments, communities from naturally disturbed or hydrologically variable habitats were more tolerant of stress than those from stable environments, suggesting that these communities may be preadapted to anthropogenic disturbances (Kaufman, 1982; Kiffney & Clements, 1996).

We do not believe that the slow recovery of the NFCC was a result of inadequate recolonization. In a long‐term assessment of four metal‐contaminated watersheds, Clements et al. (2021) reported that the fastest recovery occurred in streams where a source of recolonization was located immediately upstream. Our reference site contained a highly diverse assemblage of aquatic insects. Total abundance, number of taxa, and abundance of metal‐sensitive Heptageniidae at the reference site, located only 3.5 km upstream from BNT, are similar to, or exceed those measured in reference streams throughout Colorado (Clements et al., 2000; Schmidt et al., 2010). We believe that the nearby and productive reference site likely provided a sufficient density and diversity of organisms to adequately recolonize downstream stations. It is also unlikely that the preremediation establishment of highly competitive, novel communities downstream hindered recolonization. Total macroinvertebrate abundance at the two most contaminated sites after remediation was less than 25% of reference density, suggesting that species interactions were unlikely to have hindered recolonization.

We believe that the most likely explanation for the failure of NFCC benthic communities to recover was that the water quality in this system remained significantly degraded after remediation. Metal concentrations (as CCUs) exceeded chronic criterion levels at all downstream stations, but were especially elevated at BNT (mean post restoration CCU = 11.9). Again, this result was unexpected given that two major AMD sources were diverted around this site. Elevated metal concentrations alone might be sufficient to explain the limited recovery observed at BNT. Results of stream mesocosm experiments that exposed natural benthic communities to Gregory Incline effluents at concentrations comparable to postremediation concentrations showed significantly decreased total abundance, number of taxa, and abundance of metal‐sensitive organisms (Cadmus et al., 2016).

Elevated metal concentrations could also explain the limited recovery observed at stations located farther downstream; however, we believe that other physicochemical factors (e.g., increased concentrations of major ions) also affected recovery of macroinvertebrate communities. Spatial and temporal variation in concentrations of major ions and conductivity at BTP, Gage, and Main suggest that these patterns were associated with the water treatment facility. In contrast to patterns observed at station BNT after mining‐related effluents were diverted, conductivity and concentrations of major ions downstream of the treatment facility either changed little or increased after remediation (Meyer et al., 2022). We hypothesize that in addition to metals, elevated concentrations of major ions impeded full recovery. The toxicity of major ions can vary depending on the geochemical ion and salt mixture (Mount et al., 2019). Concentrations of SO_4_^2−^ at stations downstream of the NCCWTP were within the range of the 7‐day median effective concentrations (EC20) for inhibition of reproduction for *Ceriodaphnia dubia* reported by Mount et al. (2019). Effects of elevated major ions on macroinvertebrates are also supported by field studies in which the USEPA (1986) developed a 300‐µS/cm conductivity benchmark to protect Appalachian streams (USA) affected by coal mining operations (Cormier et al., 2013). This benchmark, which has also been supported by stream mesocosm experiments conducted using natural benthic communities (Clements & Kotalik, 2016), is designed to protect 95% of the aquatic insect species in that region. Although the benchmark was developed for the southern Appalachian Mountains, physicochemical conditions at the NFCC reference site were comparable to the naturally low‐conductivity streams in that region. After remediation, average conductivity at all NFCC sites downstream of the water treatment facility continued to exceed this benchmark and likely contributed to the inability of the benthic communities to recover to the reference condition. In particular, heptageniid mayflies, which are among the most sensitive organisms to major ions (Clements & Kotalik, 2016; Cormier et al., 2013), recovered little after remediation. In addition to these high average conductivity values, seasonal increases in conductivity during periods of low stream discharge (e.g., fall samples) at all NFCC downstream sites often exceeded 700 µS/cm (Meyer et al., 2022). These results suggest that despite significant decreases in metal concentrations after remediation, other water quality changes associated with the water treatment facility affected benthic communities.

## CONCLUSIONS

Diversion and treatment of AMD in the NFCC resulted in a rapid improvement in water quality, and benthic communities responded with increased abundance and diversity; however, elevated metal concentrations from apparent residual sources in the system continued to impact downstream benthic communities. Our results demonstrate that recovery of AMD‐impaired streams following direct AMD treatment is limited, in part, to the extent that the inputs of metals are captured and treated. In addition, treated AMD effluent still contains elevated major ion concentrations, which can act as another stressor impeding recovery. Given the near instantaneous AMD remediation treatment used in the NFCC, additional improvements in water quality are unlikely, and without additional remedial actions, we expect that downstream benthic communities will continue to be impaired. Continued water quality and biological monitoring will be needed to quantify the NFCC recovery trajectory and to inform future remediation strategies.

## Supporting Information

The Supporting Information is available on the Wiley Online Library at https://doi.org/10.1002/etc.5515.

## Disclaimer

The authors declare no conflict of interest. Any use of trade, firm, or product names is for descriptive purposes only and does not imply endorsement by the US Government.

## Author Contributions Statement

**Christopher J. Kotalik**: Conceptualization; Methodology; Formal analysis; Investigation; Data curation; Visualization; Writing—original draft; Writing—review & editing. **Joseph S. Meyer**: Conceptualization; Methodology; Formal analysis; Data curation; Writing—review & editing; Funding acquisition. **Pete Cadmus**: Conceptualization; Methodology; Investigation; Resources. **James F. Ranville**: Conceptualization; Methodology; Resources; Supervision; Project administration; Funding acquisition. **William H. Clements**: Conceptualization; Methodology; Formal analysis; Resources; Data curation; Writing—original draft; Writing—review & editing; Visualization; Supervision; Project administration; Funding acquisition.

###  

This article has earned an Open Data and an Open Materials badge for making publicly available the digitally shareable data necessary to reproduce the reported results. The data are available via the US Geological Survey ScienceBase repository at https://doi.org/10.5066/P9RQVHRO. Learn more about the Open Practices badges from the Center For Open Science: https://osf.io/tvyxz/wiki.

## Supporting information

This article includes online‐only Supporting Information.

Supplementary information.

Supplementary information.

## Data Availability

Data, associated metadata, and calculation tools are also available from the corresponding author, Christopher J. Kotalik (ckotalik@usgs.gov).
