# Supplementary material for: Integrated Assessment of Chemical and Biological Recovery After Diversion and Treatment of Acid Mine Drainage in a Rocky Mountain Stream
Source: Environ Toxicol Chem. 2022 Dec 20;42(2):512–24. doi: 10.1002/etc.5515 (PMC10108297; doi:10.1002/etc.5515)
Supplement: Supplementary file 1 — Supplementary information. [file ETC-42-512-s002.docx]

**Supplemental Data**

Integrated assessment of chemical and biological recovery after diversion and treatment of acid mine drainage in a Rocky Mountain stream

Christopher J. Kotalik^1,2^, Joseph S. Meyer^3,4^, Pete Cadmus^5^, James F. Ranville^3^, William H. Clements^2^

^1^ U.S. Geological Survey, Columbia Environmental Research Laboratory, Columbia, Missouri, USA

^2^ Department of Fish, Wildlife and Conservation Biology, Colorado State University, Fort Collins, Colorado, USA

^3^ Department of Chemistry, Colorado School of Mines, Golden, Colorado, USA

^4^ Applied Limnology Professionals LLC, Golden, Colorado, USA

^5^ Colorado Parks and Wildlife, Fort Collins, Colorado, USA

**Tables**

**Table S1.** Superfund remediation goals and State of Colorado’s stream standards for North Fork of Clear Creek (NFCC), Gilpin County, Colorado, USA, and for the mainstem of Clear Creek (CC) from the confluence with NFCC downstream to Golden, Colorado (CDPHE 2009, 2017). All concentrations are expressed as dissolved metal. For management purposes on NFCC, the high-flow period is defined as May through August; the low-flow period is September through April (CDPHE and USEPA 2004).

|  |  | Remediation goal (µg/L) | | | | |  | Stream standard (µg/L) | | | | |
| --- | --- | --- | --- | --- | --- | --- | --- | --- | --- | --- | --- | --- |
|  |  | NFCC | |  | CC | |  | NFCC | |  | CC | |
| Metal |  | High-flow | Low-flow |  | High-flow | Low-flow |  | High-flow | Low-flow |  | High-flow | Low-flow |
| Cd |  | 1.9 | 3.5 |  | 1.4 | 2.3 |  | 4.7 (1.9) ^a^ | 4.7 (3.5) ^a^ |  | 1.4 | 2.3 |
| Cu |  | 7.4 | 15.1 |  | 5.2 | 9.2 |  | 64 | 64 |  | 17 | 17 |
| Mn |  | 1,531 | 2,021 |  | 600 | 600 |  | 3,841 (1,431) ^a^ | 3,841 (2,021) ^a^ |  | 861 (600) ^a^ | 861 (600) ^a^ |
| Zn |  | 381 | 675 |  | 200 | 300 |  | 1,582 (740) ^a^ | 1,582 (740) ^a^ |  | 325 (300) ^a^ | 325 (300) ^a^ |

^a^ Primary-listed value is a temporary modification; the underlying standard is in parentheses.

**Table S2.** Sampling sites on North Fork of Clear Creek (NFCC), Gilpin County, Colorado, USA. AMD = acid mine drainage; GI = Gregory Incline; NA = not applicable; NCCWTP = North Clear Creek Water Treatment Plant; NT = National Tunnel; WWTP = Black Hawk/Central City Sanitation District Wastewater Treatment Plant.

| Site | Distance from mouth of |  |
| --- | --- | --- |
| ID | NFCC | Description |
| Ref | 13.4-14.0 | Reference: An upstream NFCC reach, starting at water-intake shed 1.4 km upstream of GI AMD input and extending 0.6 km upstream; background stream conditions unaffected by AMD. |
| BNT | 10.9 | Below National Tunnel: On NFCC, 1.1 km downstream of GI AMD input and 0.1 km downstream of NT AMD input. |
| BTP-1 | 9.8-10.0 | Below Treatment Plant: On NFCC, 2.0-2.2 km downstream of GI AMD input and 0.3-0.5 km downstream of NCCWTP. |
| BTP-2 | 5.5 | Below Treatment Plant: On NFCC, 6.5 km downstream of GI AMD input and immediately upstream of confluence from Russell Gulch, a potential source of metals when it flows. |
| Gage | 0.4 | U.S. Geological Survey gage stations (: On NFCC at USGS stream-gaging station, 11.6 km downstream of GI AMD input and 2.0 km downstream of WWTP. |
| Main | NA | On mainstem of Clear Creek, 0.4 km downstream from confluence with NFCC. |

**Table S3.** Results of least square (LS) means testing for differences in metal concentration (as cumulative criteria units, CCUs) and conductivity among sites and between restoration treatments in the North Fork of Clear Creek (CO). Only comparisons showing significant differences (p < 0.10) are included in the Table.

|  |  | Ref | BNT | BNT | BTP | BTP | Gage | Gage | Main | Main |
| --- | --- | --- | --- | --- | --- | --- | --- | --- | --- | --- |
| Metal concentration |  | (After) | (Before) | (After) | (Before) | (After) | (Before) | (After) | (Before) | (After) |
|  | Ref (Before) |  | 0.0001 | 0.0001 | 0.0001 | 0.0001 | 0.0001 |  | 0.0001 |  |
|  | Ref (After) |  | 0.0001 | 0.0001 | 0.0001 | 0.0001 | 0.0001 | 0.0008 | 0.0001 |  |
|  | BNT (Before) |  |  | 0.0001 |  | 0.0001 | 0.0001 | 0.0001 | 0.0001 | 0.0001 |
|  | BNT (After) |  |  |  | 0.0001 | 0.0001 |  | 0.0001 | 0.0001 | 0.0001 |
|  | BTP (Before) |  |  |  |  | 0.0001 | 0.0001 | 0.0001 | 0.0001 | 0.0001 |
|  | BTP (After) |  |  |  |  |  | 0.0001 | 0.0228 |  |  |
|  | Gage (Before) |  |  |  |  |  |  | 0.0001 | 0.0001 | 0.0001 |
|  | Gage (After) |  |  |  |  |  |  |  |  |  |
|  | Main (Before) |  |  |  |  |  |  |  |  |  |
|  |  |  |  |  |  |  |  |  |  |  |
|  |  |  |  |  |  |  |  |  |  |  |
| Specific conductance |  |  |  |  |  |  |  |  |  |  |
|  | Ref (Before) |  | 0.0001 | 0.0001 | 0.0001 | 0.0001 | 0.0001 | 0.0001 | 0.0001 | 0.0001 |
|  | Ref (After) |  | 0.0001 | 0.0001 | 0.0001 | 0.0001 | 0.0001 | 0.0001 | 0.0001 | 0.0001 |
|  | BNT (Before) |  |  | 0.0009 |  | 0.0030 |  | 0.0788 | 0.0046 |  |
|  | BNT (After) |  |  |  | 0.0001 | 0.0001 | 0.0001 | 0.0001 |  |  |
|  | BTP (Before) |  |  |  |  |  |  |  | 0.0001 |  |
|  | BTP (After) |  |  |  |  |  |  |  | 0.0001 | 0.0083 |
|  | Gage (Before) |  |  |  |  |  |  |  | 0.0001 |  |
|  | Gage (After) |  |  |  |  |  |  |  | 0.0001 | 0.0594 |
|  | Main (Before) |  |  |  |  |  |  |  |  |  |

**Table S4.** Results of least square (LS) means testing for differences in benthic community metrics, metal concentrations and specific conductance in the North Fork of Clear Creek (CO) across stations and seasons. Stations with the same letter are not significantly different from one another based on comparisons of LS means.

| Metric |  |  |  | Station |  |  |  |  | Season |  |
| --- | --- | --- | --- | --- | --- | --- | --- | --- | --- | --- |
|  |  | Ref | BNT | BTP | Gage | Main |  | Spring | Summer | Fall |
| Number of taxa |  | A | C | D | C | B |  | A | A | A |
| Total abundance |  | A | C | C | B | A |  | B | A | A |
| Total Heptageniidae |  | A | C | C | C | B |  | A | A | A |
| Metal concentration |  | E | A | B | C | D |  | A | A | A |
| Specific conductance |  | C | B | A | A | A |  | B | C | A |

**Table S6.** Abbreviated and full taxa description of benthic macroinvertebrate taxa listed in Figure 5.

| **Abbreviated taxa** | **Full taxa description** |
| --- | --- |
| Acentr | *Acentrella* sp. |
| Arcgra | *Arctopsyche grandis* |
| Baetis | *Baetis* sp. |
| Capnia | *Capnia* sp. |
| Chloro | Chloroperlidae |
| Cinygm | *Cinygmula* sp. |
| Diames | Diamesinae |
| Drudod | *Drunella dodsii* |
| Drugra | *Drunella grandis* |
| Epeorus | *Epeorus* sp. |
| Glosso | *Glossoma* sp. |
| Hetcor | *Heterlimnius corpulentus* |
| Hydrops | *Hydropscyche* sp. |
| Hyrdra | Hydracarina |
| Isoperla | *Isoperla* sp. |
| Lepido | *Lepidostoma* sp. |
| Megsig | *Megarcys signata* |
| Oligoc | Oligochaeta |
| Orthoc | Orthocladiinae |
| Propod | *Prostoia* sp./*Podmosta* sp. |
| Rhithr | *Rhithrogena* sp. |
| Rhyacop | *Rhyacophila* sp. |
| Simuld | *Simulium* sp. |
| Taenio | *Taenionema* sp. |
| Tanypo | Tanypodinae |
| Tanyta | Tanytarsini |
| Zapada | *Zapada* sp. |

**Figures**


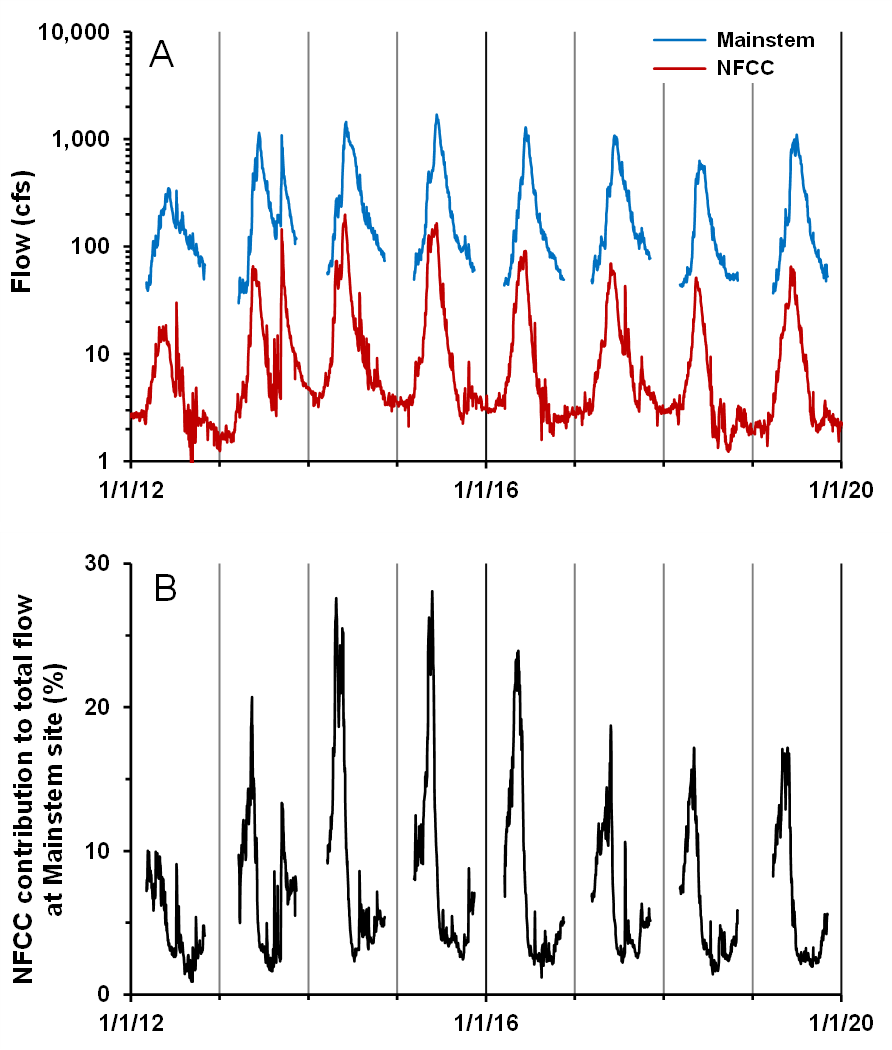


**Date**

**Figure S1.** Measured discharge at the Gage station (U.S. Geological Survey stream gage 06718550) on North Fork of Clear Creek (NFCC) from 2012 through 2019, and calculated discharge at the Mainstem site on Clear Creek, based on measured discharges at the Gage station and at the U.S. Geological Survey former-gaging station on Clear Creek above Johnson Gulch near Idaho Springs (06718300), located approximately 4 km upstream on Clear Creek from the confluence. This gage station was operated by Clear Creek Consultants, Inc. from 2009-2019.

**
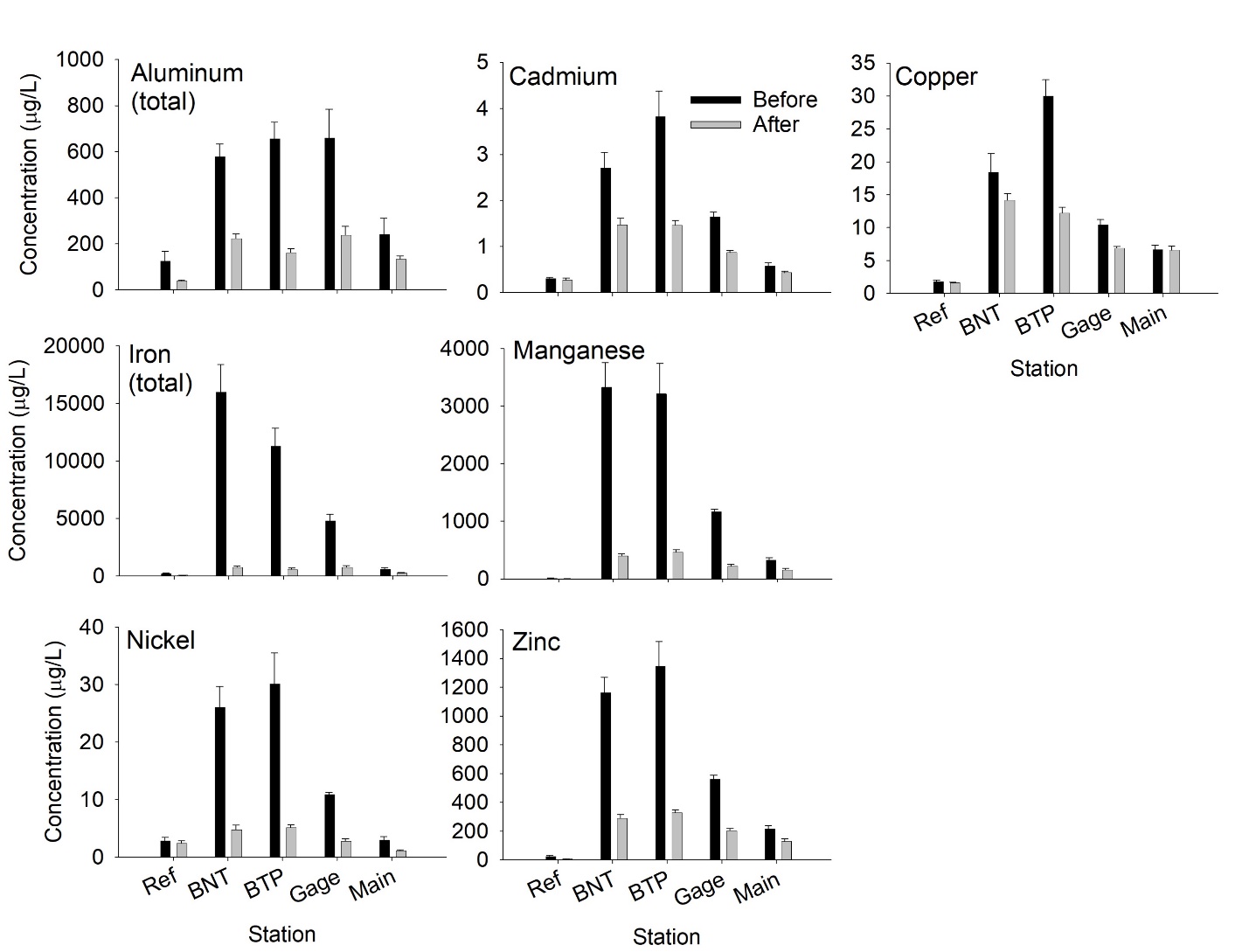
**

**Figure S2**. Spatiotemporal variation in the mean (+ s.e.) concentration of all metals that comprised the cumulative criteria unit (CCU) at North Fork of Clear Creek, showing differences among stations and between treatments (before versus after remediation).

**
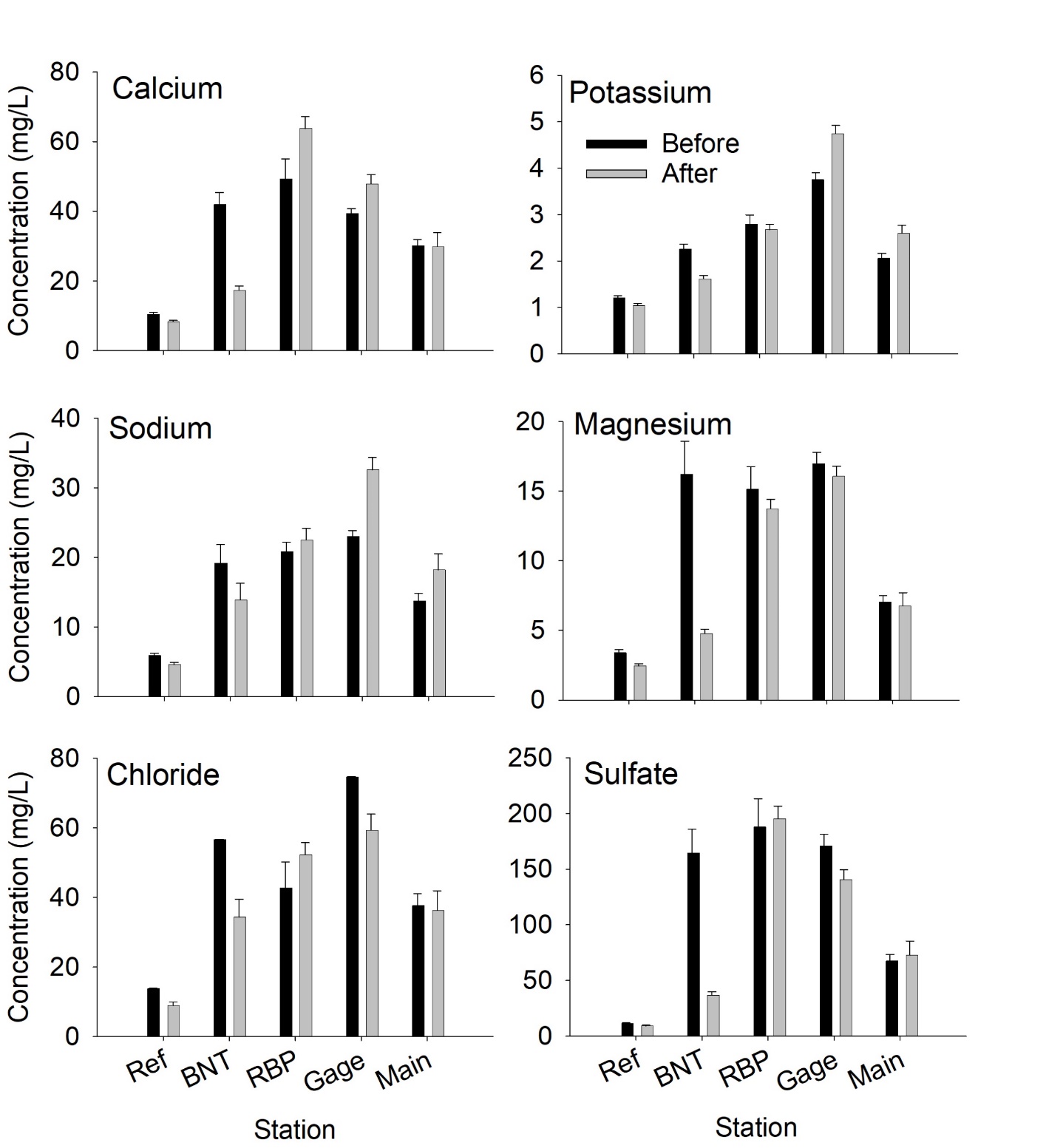
**

**Figure S3**. Spatiotemporal variation in the mean (+ s.e.) concentration of major ions in North Fork of Clear Creek (NFCC) among stations, and before versus after remediation.

**Watershed Characteristics**

Average annual precipitation in Black Hawk is 40 cm, with the highest amounts in summer and lowest amounts in winter (www.weather.com). Monthly average high temperatures range from +1 (December-January) to +24 °C (July-August), and monthly average lows range from -12 to +7 °C ([www.weather.com](http://www.weather.com)). During some winter months, the stream surface freezes.

The surrounding terrain is mountainous, with “steep-walled canyons and narrow valley floors” (CDPHE and USEPA 2004). Within the montane ecosystem in which the study area occurs, grasses, shrubs, and ponderosa pine trees grow on dry, south-facing slopes; Douglas fir, lodgepole pine, and Engelmann spruce trees grow on moister north-facing slopes; and willows and quaking aspen trees grow in moister soils (Anchor Point 2009). The underlying bedrock consists of Precambrian crystalline rocks containing gneiss, granite, schist, and pegmatite (Butler et al. 2009). The host rocks include tertiary intrusions that are the sources of sulfide bearing ores, which contain Ag, Au, Cd, Cu, Mn, Ni, Pb, and Zn (Butler et al. 2009, CDPHE 2017).

No industrial activity currently affects the North Fork of Clear Creek (NFCC). Although mining historically dominated Gilpin County, the economy is now dominated by casino gambling in Black Hawk and Central City, and by tourism and recreation throughout the county. In 2010, the population of the county was relatively low (5,441, or 14/km^2^), mostly concentrated in Black Hawk and Central City (USCB 2012).

Clear Creek upstream from our mainstem sampling site (the Main site) has received effluent from the Argo Tunnel Water Treatment Plant in the City of Idaho Springs (~11 km upstream of the Main site) since 1998, which uses a high-density sludge lime treatment system to remove metals from acid mine drainage (AMD) waters flowing out of the Argo Tunnel. Additionally, Interstate Highway 70 is adjacent to Clear Creek from its headwaters down to the junction with Highway 6 (~5.5 km upstream from the Main site). Thus, Clear Creek has received considerable road runoff, including de-icing salts during winter, since the construction of Interstate 70 was completed along this reach in the 1960s.

**Calculation of Composite Water Chemistry for Biological-sampling Dates**

The rules for calculating composite water chemistry were:

1. Spring = January to May, Summer = July through September; Fall = October and November. No biological samples were collected in June or December, and biological samples in May were always collected before the high-runoff period.

2. Chemical-sampling dates in June were not used to calculate composite chemistry for biological-sampling dates, because they would have been during high runoff of snowmelt, which would not have represented Spring or Summer exposures.

3. Sampling dates in Spring months were not used to calculate composite chemistry for biological-sampling dates in Summer months (because of the intervening high-runoff period).

4. Sampling dates in Summer months were not used to calculate composite chemistry for biological-sampling dates in Spring months (because of the intervening high-runoff period).

5. Composite chemistry for a given biological-sampling date was calculated from all eligible data (as constrained by rules 2 to 4 above) up to 90 d before and up to 7 d after the biological-sampling date. The composite value was the average of all acceptable values within that time window. We included chemistry data up to 7 d after a biological-sampling date because water-chemistry samples were not always collected on the biological-sampling date.

**Calculation of Criteria Units and Cumulative Criteria Units**

We used a slight modification of the procedure in Clements et al. (2021) to calculate the number of chronic cumulative criteria units (CCUs) for the metal mixture at each site on each biological-sampling date.

We first calculated chronic criteria units (CUs) for each of the 7 metals included in the current analysis (Al, Cd, Cu, Fe, Mn, Ni, and Zn), as CU = measured concentration/criterion. Dissolved concentrations were used for all of the measured metal concentrations except Fe, for which the U.S. Environmental Protection Agency’s (USEPA’s) water quality criterion is based on total metal concentration. The criteria we used were the same as those used by Clements et al. (2021), except for Al [for which we used the USEPA’s Excel-based Al criteria calculator] and Fe [for which we used the USEPA’s aquatic life criterion instead of the concentration of 250 μg Fe/L that Clements et al. (2021) used, based on results in Cadmus et al. (2018)]. We calculated the criteria as follows, where DOC is dissolved organic carbon concentration (mg C/L), EXP is the exponential function, and H is water hardness (mg/L as CaCO_3_), :

Al criterion = pH-, hardness-, and DOC-dependent; calculated using USEPA’s Al criteria calculator (USEPA 2021a).

Cd criterion = [1.101672 -0.041838·ln(H)]·EXP[0.7977·ln(H) – 3.909] (USEPA 2021b).

Cu criterion = EXP[0.786·ln(DOC) + 0.582·ln(H) + 0.966·pH – 8.79] (Brix et al. 2017).

Fe criterion = 1,000 μg/L (a constant value independent of water chemistry; USEPA 2021b).

Mn criterion = EXP[0.3331·ln(H) + 5.8743] (Stubblefield and Hockett 2000).

Ni criterion = 0.997·EXP[0.8460·ln(H) + 0.0584] (USEPA 2021b).

Zn criterion = 0.986·EXP[0.8473·ln(H) + 0.8840] (USEPA 2021b).

**References**

Anchor Point. 2009. Gilpin County, Colorado: Community wildfire protection plan. Report prepared for Gilpin County, Colorado by Anchor Point, Boulder, Colorado, USA. Available at: https://gilpin.extension.colostate.edu/programs/natu/fire/ .

Brix KV, DeForest DK, Tear L, Grosell M, Adams WJ. 2017. Use of multiple linear regression models for setting water quality criteria for copper: A complementary approach to the Biotic Ligand Model. *Environmental Science and Technology* 51:5182-5192.

Bulter BA, Ranville JF, Ross PE. 2009. Spatial variations in the fate and transport of metals in a mining-influenced stream, North Fork Clear Creek, Colorado. *Science of the Total Environment* 407:6223-6234.

Cadmus P, Brinkman SF, May MK. 2018. Chronic toxicity of ferric iron for North American aquatic organisms: Derivation of a chronic water quality criterion using single species and mesocosm data. *Archives of Environmental Contamination and Toxicology* 74:605-615.

CDPHE (Colorado Department of Public Health and Environment). 2017. Fifth five-year review report for Central City/Clear Creek Superfund site, Gilpin and Clear Creek Counties, Colorado. U.S. Environmental Protection Agency, Denver, Colorado, USA.

CDPHE (Colorado Department of Public Health and Environment), USEPA (United States Environmental Protection Agency). 2004. Central City/Clear Creek Superfund Site: Operable Unit 4. Record of Decision. SDMS Document ID 1051452, U.S. Environmental Protection Agency, Washington, DC, USA.

Stubblefield WA, Hockett JR. 2000. Derivation of a Colorado state manganese table value standard for the protection of aquatic life. ENSR Corporation, Fort Collins, Colorado, USA.

USCB (United States Census Bureau). 2012. Colorado: 2010. Summary population and housing characteristics. CPH-1-7. U.S. Department of Commerce, Washington, DC, USA.

USEPA (U.S. Environmental Protection Agency). 2021a. Aluminum Criteria Calculator V2.0. U.S. Environmental Protection Agency, Washington, DC, USA, Available at: <https://www.epa.gov/wqc/aquatic-life-criteria-aluminum> .

USEPA (U.S. Environmental Protection Agency). 2021b. National recommended water quality criteria. U.S. Environmental Protection Agency, Washington, DC, USA. Available at: <https://www.epa.gov/wqc/national-recommended-water-quality-criteria-aquatic-life-criteria-table> .
